# Similar Presynaptic Action Potential-Calcium Influx Coupling in Two Types of Large Mossy Fiber Terminals Innervating CA3 Pyramidal Cells and Hilar Mossy Cells

**DOI:** 10.1523/ENEURO.0017-23.2023

**Published:** 2023-02-03

**Authors:** Endre Levente Marosi, Antónia Arszovszki, János Brunner, János Szabadics

**Affiliations:** Institute of Experimental Medicine, Budapest, 1083, Hungary

**Keywords:** axonal physiology, CA2, CA3, calcium currents, dentate gyrus, hilus

## Abstract

Morphologically similar axon boutons form synaptic contacts with diverse types of postsynaptic cells. However, it is less known to what extent the local axonal excitability, presynaptic action potentials (APs), and AP-evoked calcium influx contribute to the functional diversity of synapses and neuronal activity. This is particularly interesting in synapses that contact cell types that show only subtle cellular differences but fulfill completely different physiological functions. Here, we tested these questions in two synapses that are formed by rat hippocampal granule cells (GCs) onto hilar mossy cells (MCs) and CA3 pyramidal cells, which albeit share several morphologic and synaptic properties but contribute to distinct physiological functions. We were interested in the deterministic steps of the action potential-calcium ion influx coupling as these complex modules may underlie the functional segregation between and within the two cell types. Our systematic comparison using direct axonal recordings showed that AP shapes, Ca^2+^ currents and their plasticity are indistinguishable in synapses onto these two cell types. These suggest that the complete module that couples granule cell activity to synaptic release is shared by hilar mossy cells and CA3 pyramidal cells. Thus, our findings present an outstanding example for the modular composition of distinct cell types, by which cells employ different components only for those functions that are deterministic for their specialized functions, while many of their main properties are shared.

## Significance Statement

How different neurons should be for distinct functionality? Several examples showed that distinct neuron types use identical ion channels or synaptic proteins in various combinations to achieve cell type-specific excitability or synaptic properties. But what about complete modules, in which mechanisms cooperate for fundamental functions, such as the action potential (AP)-I_Ca_ coupling that translates presynaptic activity to Ca^2+^ influx that triggers synaptic release. Do cell type-specific functions determine the operation of modules? Here we examined AP-I_Ca_ coupling in the common synaptic drive to CA3 pyramidal cells and hilar mossy cells (MCs), which contribute to distinct hippocampal functions. We revealed that their main excitatory synapses from granule cells (GCs) share all components of AP-I_Ca_ coupling, demonstrating that distinct cell types can use identical synaptic modules.

## Introduction

The parallel information processing capacity of the brain is fundamentally defined by the diversity of synaptic outputs from a single axon to various types of postsynaptic neurons. Individual axons evoke diverse synaptic effects on different target neurons, and the synaptic properties depend on the identities of both the presynaptic and postsynaptic cells (Nusser, 2018). Single spikes can trigger specific synaptic machineries, which are characterized by connection-specific release properties and initial strengths. Furthermore, synaptic responses show facilitation or depression during repetitive presynaptic activity depending on the short-term plasticity mechanisms of the particular type of synapse. Both the reliability and the short-term plasticity of the synapses strongly depend on the coupling between the action potential and the calcium influx (AP-I_Ca_ coupling), as it defines how release mechanisms perceive the propagating presynaptic action potentials (Katz and Miledi, 1967, 1970). The AP-I_Ca_ coupling is complex module composed by multiple independent mechanisms, such as local excitability, active conductance underlying APs and Ca^2+^ currents, and each of these can specifically contribute to synaptic release. Therefore, AP-I_Ca_ coupling is critical in synapse-specific diversification, and it contributes to the physiological functions of the participating cell types. The assembly of synaptic machinery is defined by transsynaptic pairing of cell adhesion molecule sets, whose expression is different in distinct cell types (de Wit and Ghosh, 2016). Thus, cell type identity and cell type-specific synaptic functions are interdependent, and they define specific routing of information in parallel circuits (Buonomano, 2000; Pouille and Scanziani, 2004).

Mossy fiber (MF) synapses, formed by the axons of dentate gyrus (DG) granule cells (GCs) also shows variable target-cell-dependent reliability and short-term plasticity (Toth et al., 2000; Mori et al., 2004; Szabadics and Soltesz, 2009). Notably, MFs form characteristic giant boutons (MFB) that innervate pyramidal cells in the CA3 region (CA3PCs). MFs also have numerous regularly sized terminals, which specifically target GABAergic cells (Amaral, 1978; Acsády et al., 1998), in which calcium dynamics are different (Pelkey et al., 2006). MFs also form synapses in the hilus region and hilar mossy cells (MCs) are also innervated by synapses that have unusually large volume and numerous release sites similarly to CA3PCs. Although CA3PCs and MCs share several fundamental features, their functional contribution to the hippocampal network is unique. Specifically, MCs can sufficiently control epileptic seizures (Santhakumar et al., 2005; Bui et al., 2018) and interfere with anxiety behavior (Jinde et al., 2012; Wang et al., 2021); and as their activity is sensitive to small differences in the environment, they play a key role in pattern separation of spatial and episodic information (Jinde et al., 2012; Danielson et al., 2017; GoodSmith et al., 2017; Senzai and Buzsáki, 2017; Fredes et al., 2021). In contrast, CA3PCs contribute to pattern completion by recalling complete memories from partial cues in their recurrent excitatory network (Guzman et al., 2016; Lee et al., 2020). The distinct physiological functions of MCs and CA3PCs are not surprising owing to some of their distinct morphologic, cellular and network organizational properties (Scharfman, 2016). Notably, in contrast to the recurrent network between CA3PCs, the main output of MCs is an excitatory feedback to DG granule cells, which extends the entire axis of both ipsilateral and contralateral hippocampi (Frotscher et al., 1991; Scharfman, 2016; Abdulmajeed et al., 2022). Thus, CA3PCs and MCs exert excitatory effects on distinct neuronal populations and the origin of some of their synaptic inputs is also different. In addition to their functional differences, MCs and CA3PCs express different genes, including cell-adhesion molecule sets (Cembrowski et al., 2016; Luo et al., 2021, 2022) that usually contribute to the determination of cell type identity by allowing the formation of selected synaptic inputs.

However, MCs and CA3PCs share several fundamental morphologic and cellular features. A similar set of ionic channels (Cembrowski et al., 2016) generate similar somatic firing properties (Scharfman and Schwartzkroin, 1988). In addition to typical dendritic spines, both MCs and CA3PCs form complex branching spines (called thorny excrescences) on their proximal dendrites (Amaral, 1978; Frotscher et al., 1991; Buckmaster et al., 1996; Scharfman, 2016). The specialized MF synapses that terminate on these complex spines incorporate a large number of release sites on both CA3PCs and MCs (Blackstad and Kjaerheim, 1961; Claiborne et al., 1986; Rollenhagen and Lubke, 2010), whose initial release probability is very low and show strong frequency-dependent facilitation during repetitive presynaptic activities (Toth et al., 2000; Lysetskiy et al., 2005; Drakew et al., 2017). Because of these shared properties MCs are initially considered as “modified pyramidal cells” in the hilus (Blackstad and Kjaerheim, 1961).

Albeit MCs and CA3PCs are closely related cell types, their contribution to hippocampal functions are distinct. Likewise, it is not yet clear to what extent they diverged to enable different network functions. Furthermore, within the CA3 region, the MF-evoked synaptic responses show a proximo-distal gradient, with stronger responses closer to the DG region and weaker responses toward the CA2 (Sun et al., 2017a). It is not clear whether differences in excitability and AP-related calcium signaling of presynaptic axons contribute to this functional gradient. Therefore, here, we addressed the questions whether the AP-I_Ca_ coupling is different in MFBs contacting MCs and CA3PCs, either because of the different distances between the soma and MFBs onto CA3PCs or MCs (Strüber et al., 2015; Sun et al., 2017a) or slightly different bouton sizes (Claiborne et al., 1986), and whether different AP-I_Ca_ coupling can contribute to subregion-specific outputs of MFs (Sun et al., 2017a) or distance-dependent functional division within CA3 (Lee et al., 2020). We compared the passive and active electrical properties, the Ca^2+^ currents and the AP dynamics of two populations: MFBs in the hilus onto MCs (hilMFBs), and onto PCs in the hippocampus (caMFBs), using direct patch clamp recordings from anatomically identified axons. The results revealed that all elements of the AP-I_Ca_ coupling modules are indistinguishable in hilMFBs and caMFBs, which finding has fundamental implications in the determination of cell type identities.

## Materials and Methods

### Recording conditions and solutions

Wistar rats (23–35 d old, both sexes) were deeply anesthetized with isoflurane, and 350 μm slices were prepared from the intermediate region of the hippocampus in ice-cold artificial CSF (ACSF; 85 mm NaCl, 75 mm sucrose, 2.5 mm KCl, 25 mm glucose, 1.25 mm NaH_2_PO_4_, 4 mm MgCl_2_, 0.5 mm CaCl_2_, and 24 mm NaHCO_3_, Leica VT1200 vibratome). Slices were initially incubated at 33°C for 60 min, and were then placed at room temperature until used at the time of the recordings. Recordings were performed in carbogen (95% O_2_ and 5% CO_2_) saturated ACSF solution (126 mm NaCl, 2.5 mm KCl, 26 mm NaHCO_3_, 2 mm CaCl_2_, 2 mm MgCl_2_, 1.25 mm NaH_2_PO_4_, and 10 mm glucose) at 35°C. An upright microscope (Eclipse FN-1; Nikon) equipped with infrared (900 nm) Nomarski differential interference contrast optics (25× NA1.1W objective, Nikon) was used to visualize large mossy fiber terminals. Electrophysiological data were collected using MultiClamp700B amplifier, Digidata 1440 A/D converter (250-kHz sampling rate) and pCLAMP 10.7 software (Molecular Devices). Recording pipettes were pulled from borosilicate capillaries (inner diameter: 0.75 mm, outer diameter: 1.5 mm, pipette resistance: 8–12 MΩ).

To record the membrane properties and action potentials an intracellular solution was used containing 90 mm K-gluconate, 43.5 mm KCl, 1.8 mm NaCl, 1.7 mm MgCl_2_, 0.05 mm EGTA, 10 mm HEPES, 2 mm Mg-ATP, 0.4 mm Na_2_-GTP, 10 mm phosphocreatine, and 8 mm biocytin (pH 7.25; 280–300 mOsm). Bridge balance and pipette capacitance compensation were applied in current clamp recordings. Only anatomically identified mossy fibers were included in the analysis.

### Ca^2+^ current measurement

To isolate Ca^2+^ currents, 1 μm tetrodotoxin (TTX) and 5 mm 4-aminopyridine (4-AP) were added to the standard ACSF, while in the intracellular solution K^+^ was replaced with Cs^+^, and higher EGTA concentration (5 mm) was used to minimize the run-down. During these voltage clamp experiments the membrane potential was held at −80 mV. The recorded current traces were leak-subtracted online (eight subpulses with opposite polarity to test waveform). For current–voltage relationship measurements, incremental (10 mV) voltage steps (3 ms) were applied between −60 and 40 mV. The intertrial interval was 10 s. The tail Ca^2+^ currents (at step back to −80 mV) were analyzed after three voltage steps (to 0, 30, 60 mV with 20-ms interstep intervals). As these steps activated similar macroscopic tail currents (i.e., because of the same driving force), their quantitative measurements were averaged. For AP-triggered Ca^2+^ current measurements, native AP waveforms were obtained from a whole-bouton recorded caMFB, in which propagating APs were elicited by distal stimulation (100 APs were elicited at 40 Hz with a bipolar theta glass stimulation) in the stratum lucidum. The obtained AP waveforms were embedded in a voltage command trace in four different ways: (1) 1st AP of the original waveform from −80 mV, (2) 100th AP from −70 mV, (3) 100th AP from −80 mV, (4) a burst of the initial six APs. We used online leak-subtraction with four subpulses. Series resistance was compensated (50–70% correction, 10-kHz bandwidth) and Ca^2+^ currents were recorded with 20-kHz lowpass online Bessel filter. Recordings were excluded from the analysis if the Ca^2+^ current charge declined below the 75% of the average of the first five traces. Data were quantified on average traces with additional 6-kHz offline Bessel filtering.

### Pharmacology

For rapid drug application (Fig. 4), large puff pipettes (1- to 2-MΩ resistance) were filled with either ω-Conotoxin GVIA (CoTx; 20 μm) or ω-Agatoxin IVA (AgaTx; 10 μm) containing ACSF, which also included TTX and 4-AP. These pipettes were positioned at the surface of the slice within 25–50 μm of the recorded structure, and gentle positive pressure was applied to initiate continuous drug flow. Positive pressure was continuously maintained and the stability of the flow was monitored by measuring electrical resistance. To prevent leakage, gentle suction was applied before the drug administration and the pipette was pulled away from the slice by a few hundreds of micrometers. To account for the run-down of Ca^2+^ currents (Brunner and Szabadics, 2016), we measured run-down in control experiments, where only TTX and 4-AP containing ACSF was applied and these results were subtracted from the population data. These recordings also proved that these application conditions did not affect the measurement, while they allowed rapid toxin application. In those experiments where the aim was the complete blockade of both P/Q and N-type channels during action potentials (Fig. 3*E*), CoTx (1 μm) and AgaTx (0.5 μm) was dissolved in the recording solution (Li et al., 2007), which was circulated as standard solutions during the patching of the MFBs. Bovine serum albumin (BSA) was added to this solution in 1 mg/ml concentration to prevent the nonspecific binding. CoTx, AgaTx, and TTX were purchased from Alomone Labs, 4-AP and BSA were from Sigma.

### Morphologic identification and immunolabelling

Only those recordings were considered in the analyses, which were identified as MFB in the subsequent morphologic analysis. The criteria for MFBs included sporadic presence of large boutons, which emanate filopodia, and the axons had no (CA3) or few (hilus) major branches (Blackstad and Kjaerheim, 1961; Claiborne et al., 1986; Acsády et al., 1998; Rollenhagen and Lubke, 2010). After the experiments, slices were placed into fixative solution (0.1 m PBS, 2% paraformaldehyde, 0.1% picric acid) overnight at 4°C. Afterwards the specimens were resected at 40 or 65 μm. For immunohistochemistry, the slices were washed, blocked (50 min, 0.3% Triton X-100-TBS, 10% NHS, pH 7.4) and incubated with primary antibodies against PCP4 (rabbit anti-PCP4, Novus Biologicals, 1:400, in TBS and 0,5% NHS with 0.05% azide) for 72 h. Secondary antibodies were applied overnight after washes (Alexa Fluor 488-conjugated donkey anti-rabbit IgG, Alexa 594-conjugated streptavidin, in 1:500 concentration). Sections were mounted after several washing steps using Prolong-glass medium. The recorded structures were visualized using epi-fluorescent (Leica DM 2500) or confocal (Nikon C2plus) microscopes.

In addition to the *post hoc* investigations, in some of the recordings MF identity was also tested in situ by adding Alexa Fluor 594 dye in the intracellular solution, which was imaged right after the recordings using confocal imaging (Nikon C1) in the recording microscope.

### Experimental design and statistical tests

The distance of the recorded MFBs from the proximal end of the CA3c was measured with ImageJ software following the curvature of stratum lucidum. Average data are presented as mean ± SEM and SD. Shapiro–Wilk test was used to test normality. To compare data from two groups, we used Student’s *t* test (equal variance assumed). All statistical analysis was conducted using OriginPro 2018 (OriginLab). Only those recordings were considered in the analyses, which were identified as MFB in the subsequent morphologic analysis (see above).

The membrane capacitance was calculated from the integral area of the fast component of capacitive current in response to −10 mV depolarizing voltage step (0.162 ms from the onset of the step). This area value was divided by the voltage change to obtain capacitance. To eliminate the contamination caused by the residual pipette capacitance, traces recorded in on-cell configuration before break-in were subtracted from the whole-bouton responses. The input resistance (R_in_) was calculated from the steady-state current amplitude during the −10-mV voltage step. To eliminate the contamination caused by the residual pipette capacitance, traces recorded in on-cell configuration before breaking-in were subtracted from the whole-bouton responses. Membrane time constant was measured in independent current clamp experiments by fitting the voltage response to −5 pA hyperpolarizing step with mono-exponential function.

AP parameters were quantified from APs which were evoked by long, small amplitude current injections. AP threshold was measured as the absolute voltage value at 20 V/s of the first derivative. AP amplitude was measured between the threshold and peak voltages. For AP half-width measurement, we used these amplitude values. Amplitude of the afterhyperpolarization (AHP) was measured from threshold. To measure activity-dependent plasticity of APs, repetitive firing was elicited using 100 short, large amplitude current stimuli (3 ms, 40–160 pA) at various frequencies (10, 20, 40, 80, 160 Hz). The half activation values of Ca^2+^ currents were determined by Boltzman fits (“BoltzIV” function in Origin software) on the average data without weighting.

## Results

### Recording of MFBs in the hilus and CA3 regions

**Figure 1.**
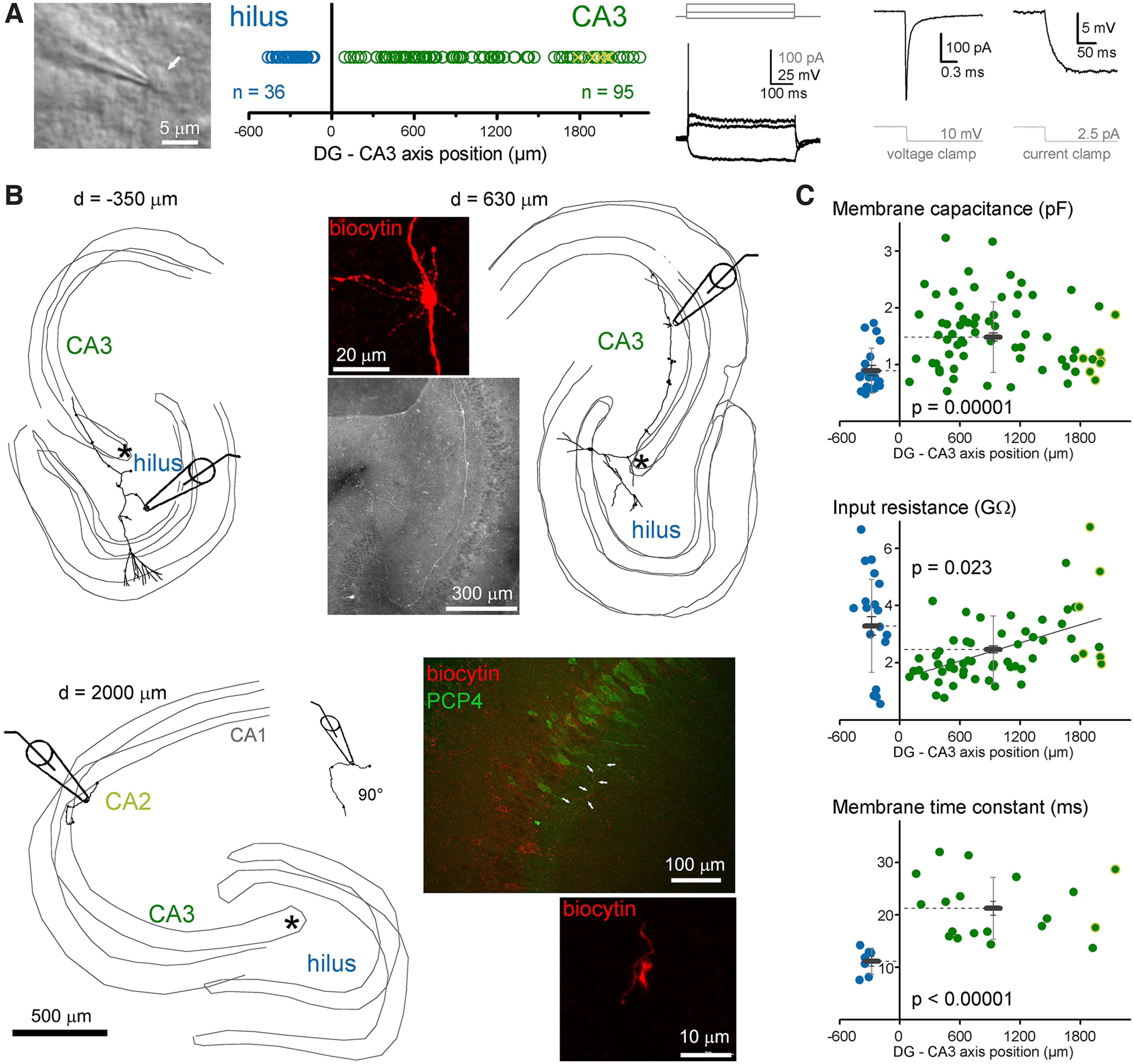
Characterization of MFBs in the hilar and CA3 areas. ***A***, MFBs were identified using Nomarski DIC imaging in acute rat slices along the entire length of mossy fiber termination zone within the hilus and CA3/CA2 regions. During the recordings, structures were considered as potential MFBs based on typical electrophysiological properties, including (from left to right) single APs during long depolarization, fast membrane transients and small steady state currents, and large voltage responses to small current injection. ***B***, All analyzed MFBs were verified using *post hoc* morphologic identification. The criteria include single major collateral in the CA3 that run mostly in the stratum lucidum and the presence of large boutons with filopodia (see inset images). Thin gray lines indicate the border of cell layers in three dimensions (up to 350 μm of the acute slice). The relative positions (d) of the recorded MFBs were measured from the starting point of the CA3 cell layer, which is marked as asterisks on these three examples. Numbers next to the reconstructions indicate the measured position of the recorded MFB. For the most distal recordings, we tested the target area with the CA2 regional marker, PCP4 (Kohara et al., 2014), using immunohistochemistry. Arrows on the example PCP4 image show a biocytin-labeled MF and a typical MFB of this axon is also shown. ***C***, Passive membrane properties of hilMFBs (blue) and caMFBs (green). Each dot represents individual recording at their distance from the starting point of the CA3 area, with hilMFBs in the negative directions, whereas caMFBs are distributed along positive *x*-axis. Green symbols with yellow edges indicate caMFBs within the PCP4-labeled area. Gray bars show the average for hilMFBs and caMFBs with SD (light gray) and SEs (gray).

To compare their excitability and calcium-signaling properties, MFBs in CA3 and hilar regions were selected for recording using Nomarski DIC image in acute rat hippocampal slices (Fig. 1*A*). Only large boutons were selected, which are known to target CA3PCs or MCs. In addition to the regional comparison, we also considered the relative position of the MFB by measuring the recording sites from the most proximal point of the CA3 stratum lucidum, where the pyramidal layer is formed. This measurement considered the curvature of the CA3 layers. The identity of all recorded MFBs were confirmed by morphologic criteria checks (see Materials and Methods). To avoid potential influence of cut end axons and damaged morphology, only those MFBs were included in the analysis that were found on at least 500-μm axonal segments and the recording site was not in the vicinity of the cut end of the axon. All MFBs were identified and a few of them were also 3D reconstructed for presentation purposes (Fig. 1*B*). Altogether, 36 hilMFBs and 95 caMFBs met the morphologic criteria. The parent soma of the recorded boutons also recovered in some cases (hilMFBs: 12, caMFBs: 25). We also checked whether the most distally recorded MFBs that were located in the CA2 area denoted by PCP4-stained pyramidal cells (Zhao et al., 2001; Kohara et al., 2014). However, not all distal MFBs were tested with PCP4-staining. We identified eight of the recorded boutons within the PCP4+ region. Their properties were not distinguishable from CA3 boutons. Therefore, these data were pooled and for simplicity we referred to them collectively as caMFBs but individual data points from these boutons are marked on the figures.

### Variation of passive membrane properties according to the target area and the position of MFBs

First, we investigated the passive membrane properties of MFBs. The membrane capacitance of the hilMFBs was smaller than that of the caMFBs (0.896 ± 0.089 and 1.495 ± 0.073 pF, *n* = 20 and 73, *p* = 0.023, *t*_(31.01)_ = −2.38; Fig. 1*C*). Furthermore, at the population level, the input resistance of hilMFBs was larger than that of caMFBs (3.29 ± 0.32 and 2.46 ± 0.12 GΩ, *n* = 25 and 93, *p* = 0.023, *t*_(31.0)_ = −2.38). In addition to this region-specific difference, a clear tendency was observed within the CA3. The most proximal caMFBs showed the smallest, whereas the most distal caMFBs showed larger input resistance (linear fit, *p* = 0.0005, *t* = 4.587, Pearson’s *R*: 0.522). Some of caMFBs identified in CA2 region showed large R_in_, consistently with their smaller size (Kohara et al., 2014). The smaller capacitance of the hilMFBs reflect the smaller size of these boutons compared with caMFBs (Claiborne et al., 1986) because the short time window of this measurement (0.162 ms) integrates only local membranes. In contrast, measurements of input resistance consider larger axon segments and, therefore, reflect the membranes, not only in the boutons but also in their vicinity. The independent measurement of the membrane time constant in current clamp recordings also revealed significant differences between hilMFBs and caMFBs (13.45 ± 1.48 and 20.33 ± 1.55 ms, *n* = 7 and 19, *p* < 0.00,001, *t*_(23.3)_ = 6.13). However, the membrane time constant of caMFBs did not show any significant correlation to the location within the CA3 region. The resting membrane potential was similar in hilMFBs and caMFBs (−64.5 ± 1.2 and −66.9 ± 1.0 mV, *n* = 7 and 17, *p* = 0.18, *t*_(22)_ = 1.37). As different passive membrane properties influence active signaling, next we compared the spiking properties of hilMFBs and caMFBs.

### The action potential kinetics are similar in caMFBs and hilMFBs

**Figure 2.**
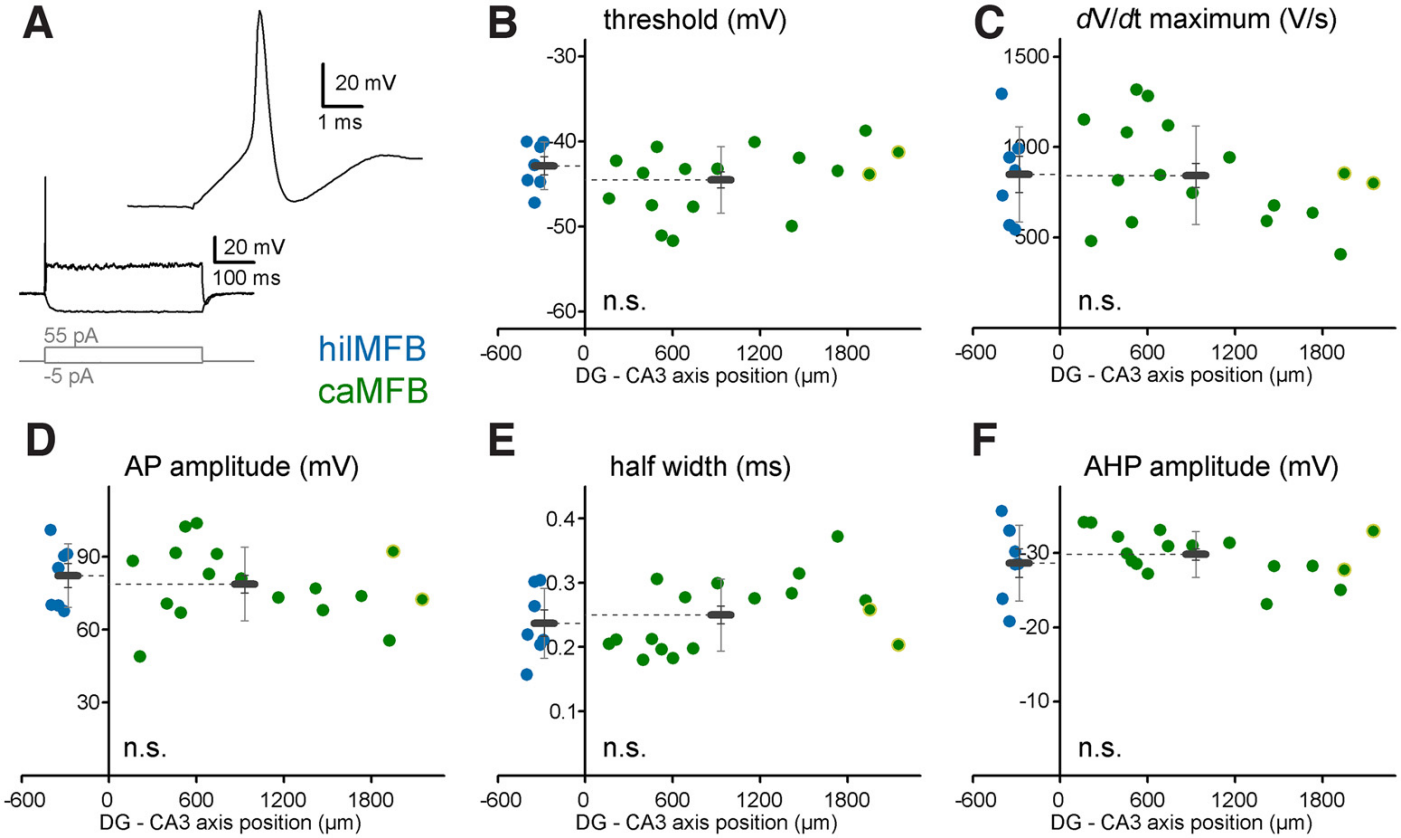
Action potentials in caMFBs and hilMFBs. ***A***, MFBs were stimulated with long current injections, which elicited single APs at the onset of the depolarization. ***B***, AP threshold of hilMFBs (blue) and caMFBs (green), measured as the voltage at the 20 V/s value of the first derivative. Each colored dot represents individual recordings at their distance from the starting point of the CA3 area, with hilMFBs with negative directions whereas caMFBs are distributed along positive *x*-axis values. Bars show the average for hilMFBs and caMFBs with SEM and SD. Green symbols with yellow circles indicate caMFBs within PCP4-labeled, CA2 area. Panels below use the same markings. ***C***, The maximum acceleration of the rising phase of the APs was measured as the peak of the derivative of the AP. ***D***, The amplitude was measured between the threshold and peak of the APs. ***E***, Width of APs was measured at the half amplitude. ***F***, Amplitude of the afterhyperpolarization (AHP) was measured from the threshold. Values were compared with Student’s *t* test. Note that none of the AP parameters were different between caMFBs and hilMFBs.

Action potentials (APs) were evoked in MFBs using long, small amplitude current injections (500 ms, 20–100 pA; Fig. 2*A*). Both hilMFBs and caMFBs responded typically with one or two APs at the onset of the depolarizations, similarly as reported before for caMFBs (Geiger and Jonas, 2000). The APs were elicited at similar threshold (hilMFB: −42.8 ± 1 mV, caMFB: −44.5 ± 0.9 mV, *t*_(22)_ = −1.024, *p* =0.317, *n* = 7 and 17; Fig. 2*B*), accelerated similarly (hilMFB: 849 ± 99 V/s, caMFB: 844 ± 66 V/s, *t*_(22)_ = −0.043, *p* =0.966, *n* = 7 and 17; Fig. 2*C*) and reached comparable amplitude from threshold (hilMFB: 82.2 ± 4.9 mV, caMFB: 78.9 ± 3.7 mV, *t*_(22)_ = −0.522, *p* = 0.607, *n* = 7 and 17; Fig. 2*D*). Furthermore, the width of the APs, which is critical for the Ca^2+^ influx (Bischofberger et al., 2002; Brunner and Szabadics, 2016) was also similarly narrow in hilMFBs and caMFBs (hilMFB: 0.237 ± 0.021 ms, caMFB: 0.25 ± 0.014 ms, *t*_(22)_ = 0.518, *p* = 0.61, *n* = 7 and 17; Fig. 2*E*). The amplitude of the after-hyperpolarization of the APs were also similar (hilMFB: −28.7 ± 1.9 mV, caMFB: −29.8 ± 0.8 mV, *t*_(22)_ = −0.694, *p* = 0.495, *n* = 7 and 17; Fig. 2*F*). We did not correlate these parameters with the relative position of caMFBs because of the insufficiently small numbers of measurements. It is worth noting that, these AP parameters were also similar to previously reported values from MFBs in the CA3 area (Geiger and Jonas, 2000; Alle et al., 2009). Altogether, these recordings show that the shapes, kinetics and amplitudes of the APs are consistent in caMFBs and hilMFBs.

### Calcium currents in hilMFBs and caMFBs

**Figure 3.**
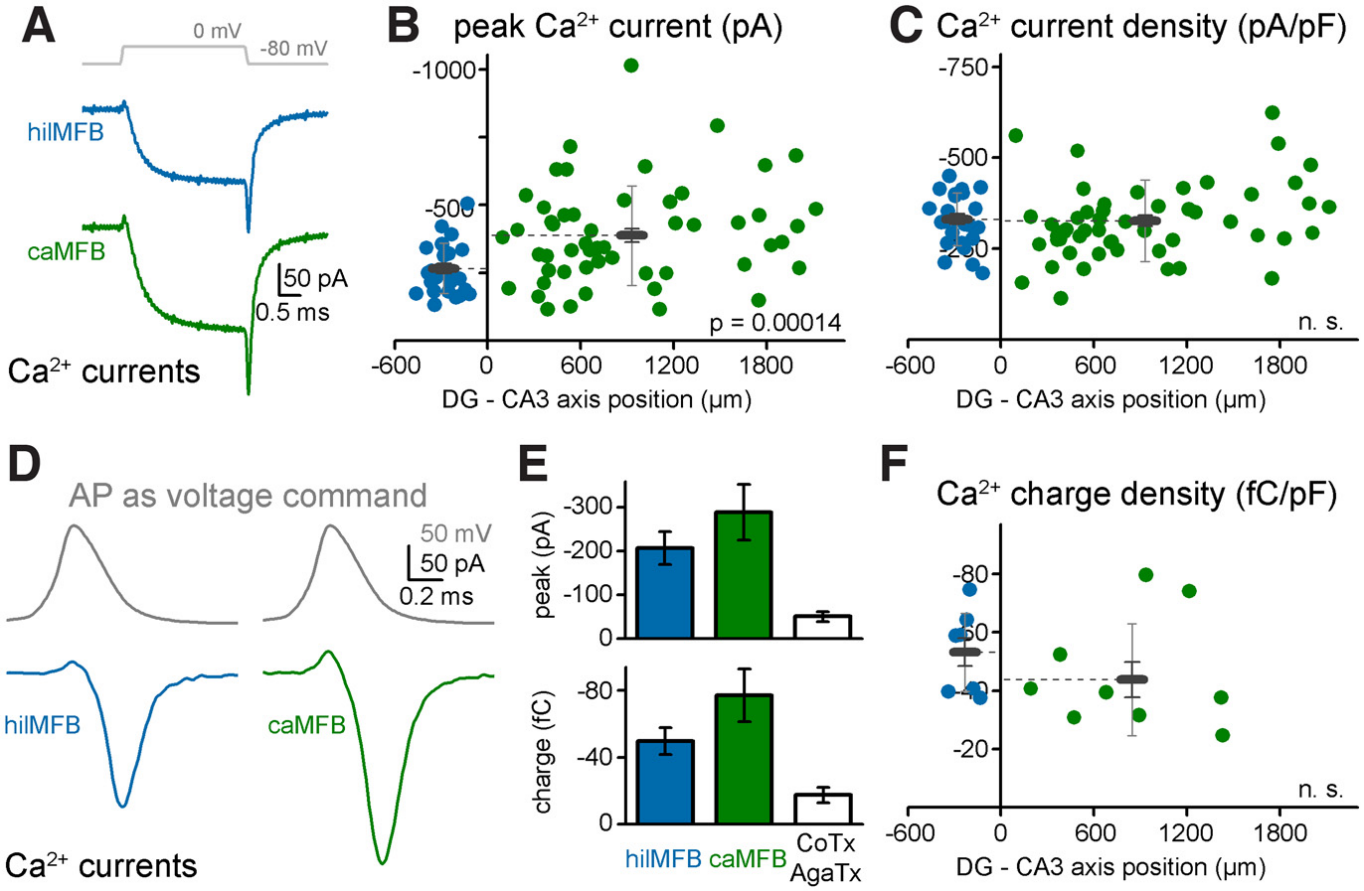
Ca^2+^ currents in caMFBs and hilMFBs. ***A***, Average Ca^2+^ current traces evoked by conventional voltage steps to 0 mV from all hilMFB and caMFB recordings. ***B***, Peak amplitudes of the tail currents from individual MFBs and their average. ***C***, To calculate Ca^2+^ current densities in individual MFBs, peak current was divided by their membrane capacitance. Notice that in contrast to the absolute peaks, the Ca^2+^ current densities were not different in hilMFBs and caMFBs. ***D***, To directly measure Ca^2+^ currents during native axonal APs, we obtained propagating AP waveform from an MFB in the CA3 area while the DG was stimulated with an extracellular electrode. This AP was employed as a voltage waveform in voltage clamp recordings. Traces show averages from all recordings. ***E***, Similarly to square pulses, native AP waveforms evoked larger Ca^2+^ currents in caMFBs compared with hilMFBs as measured either as the peak or the integral area (charge) of the currents. Columns show average and SEM. The third column shows the currents recorded in the presence of N-type and P/Q-type Ca^2+^ channel inhibitors, 1 μm Conotoxin-GVIA (CoTx) and 0.5 μm Agatoxin-IVa (AgaTx) in the bath. ***F***, Similarly to square pulses, when the measured Ca^2+^ currents were normalized by the membrane surface (capacitance) of the individual boutons, hilMFBs and caMFBs were not different.

The next critical step in the coupling of presynaptic activity to synaptic release is the AP-evoked Ca^2+^ influx. To investigate possible region-specific differences in this process, first we characterized axonal Ca^2+^ currents in the two populations of MFBs using conventional voltage steps (Fig. 3*A*). The pharmacologically isolated Ca^2+^ currents (Fig. 3*A*, in the presence of TTX and 4-AP) showed typical kinetic and voltage-dependence characteristics both in hilMFBs and caMFBs (Bischofberger et al., 2002; Li et al., 2007). However, the observed tail Ca^2+^ currents in hilMFBs were smaller than in caMFBs (hilMFBs: −267 ± 18 pA, *n* = 26; caMFBs: −389 ± 24 pA *n* = 57 caMFBs, *t*_(81)_ = −3.185, *p* = 0.002; Fig. 3*B*). This difference in the Ca^2+^ current amplitude in hilMFBs and caMFBs is consistent with the difference in their sizes (Claiborne et al., 1986), which was also observed in our local membrane capacitance measurements (Fig. 1*C*). Indeed, current density, which is calculated for each MFB as the ratio of the peak Ca^2+^ current and local membrane capacitance of the recorded structure, was similar in the two populations (hilMFBs: −331 ± 14 pA/pF; caMFBs: −326 ± 15 pA/pF, *t*_(81)_ = 0.192, *p* = 0.848; Fig. 3*C*). These observations suggest that albeit the total Ca^2+^ current is larger in caMFBs, the Ca^2+^ influx at individual release sites is similar in the two regions. Note that each MFBs incorporate a large number of release sites in which Ca^2+^ signaling is relatively autonomous (Chamberland et al., 2018). In addition to similar Ca^2+^ current densities in hilMFBs and caMFBs, their activation and deactivation kinetics were also similar (time constants of activation at 0 mV: 0.353 ± 0.019 and 0.329 ± 0.016 ms, *t*_(61)_ = 0.7, *p* = 0.49, and deactivation from 0 to −80 mV: 0.083 ± 0.015 and 0.082 ± 0.005 ms *t*_(61)_ = 0.027, *p* = 0.98, *n* = 12 and 51, respectively), similarly to previous reports on CA3 MFBs (Bischofberger et al., 2002; Li et al., 2007). We did not detect a correlation in the Ca^2+^ current density to the location of the bouton within the CA3 region.

Next, we tested Ca^2+^ currents elicited by a naturally propagating AP voltage waveform, which was recorded in a caMFB during distal extracellular stimulation (see Materials and Methods; Fig. 3*D*). Similar to voltage step protocols, the amplitude of AP-evoked Ca^2+^ currents were somewhat smaller in hilMFBs compared with caMFBs (−218 ± 41, −306 ± 68 pA, *t*_(14)_ = 1.013, *p* = 0.328, *n* = 7 and 9; Fig. 3*E*), however, because of the larger variance in these measurements the difference was not significant statistically. The kinetics of the AP waveform-evoked Ca^2+^ currents followed the expected kinetics in both cases: started after the rising phase of the AP and had a peak during the repolarization phase when the driving force is large and the proportion of activated channels is high. Therefore, the peak amplitude of AP-elicited Ca^2+^ current depends on various additional parameters as it is determined by constantly decaying voltage. To account for these additional variabilities, we also compared the charge of the evoked Ca^2+^ currents, which reflects the amount of the ions that enter the boutons (hilMFBs: −51.9 ± 8.8 fC, caMFBs: −82 ± 16.7 fC, *t*_(14)_ = 1.462, *p* = 0.166, *n* = 7 and 9; Fig. 3*E*). When this value was normalized to the membrane capacitance (indicative of the membrane surface), similarly to conventional voltage steps, the native propagating AP waveform elicit similar Ca^2+^ influx in hilMFBs and caMFBs (−53.5 ± 5.4, −45.7 ± 6.4 fC/pF, *t*_(14)_ = −0.901, *p* = 0.383, *n* = 7 and 9; Fig. 3*F*). It was shown previously that the majority of Ca^2+^ currents are mediated by two channel types in MFBs, the P/Q-type and N-type Ca^2+^ channels (Li et al., 2007). Our results confirmed the major contribution, as AP-evoked currents were minimal in the presence of specific P/Q-type and N-type channel inhibitors, Agatoxin-IVA and Conotoxin-GVIA (peak: −40 ± 5 pA, charge: −17.5 ± 2.2 fC, *n* = 4; 2 caMFBs and 2 hilMFBs; Fig. 3*E*).

Altogether these results suggest that not only the AP shape but the subsequent Ca^2+^ influx is also similar in caMFBs and hilMFBs.

### Components of Ca^2+^ currents in hilMFBs and caMFBs

MFBs in the CA3 region use two main types of Ca^2+^ channels that allow different modulation and release kinetics (Pelkey et al., 2006; Li et al., 2007; Chamberland et al., 2017). P/Q-type channels are efficiently activated by APs, while N-type activation is less efficient (Li et al., 2007) and allows for various forms of presynaptic modulation (Toth et al., 2000; Pelkey et al., 2008; Brunner et al., 2013). Therefore, next we determined to what extent each Ca^2+^ channel subtype contributes to the total presynaptic Ca^2+^ currents in hilMFBs and caMFBs using specific peptide toxins. Conotoxin-GVIA specifically inhibits N-type channels, whereas Agatoxin-IVA blocks P/Q-type channels.

**Figure 4.**
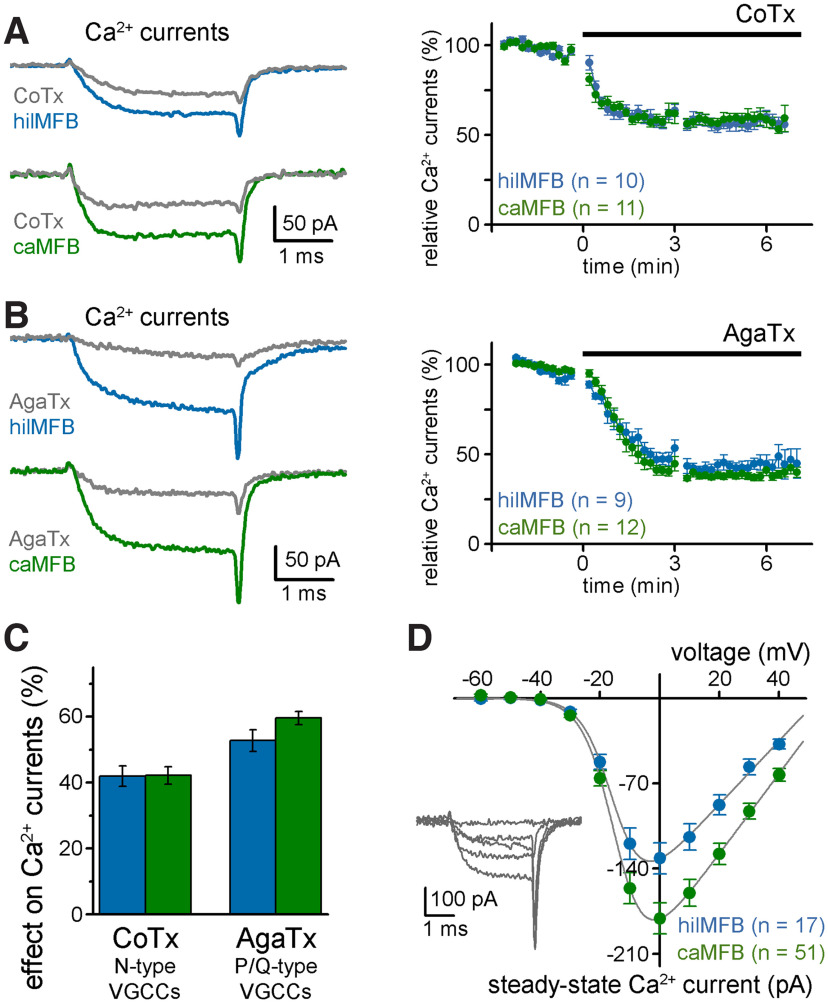
Similar Ca^2+^ current components in caMFBs and hilMFBs. ***A***, N-type-specific Ca^2+^ channel blocker, *ω-*Conotoxin-GVIA (CoTX; locally applied) substantially inhibited the isolated Ca^2+^ currents as shown in two representative recordings from a hilMFB and a caMFB (left) and the average effects (right). ***B***, P/Q-type Ca^2+^ channels specific blocker, *ω-*Agatoxin-IVA (AgaTx; locally applied) induced an even greater inhibition of the Ca^2+^ currents of MFBs. ***C***, Summary graph showing the average proportions of Ca^2+^ current inhibited by CoTX and AgaTx in hilMFBs and caMFBs. Both toxins had similar effects on hilMFBs and caMFBs. ***D***, Similar voltage dependence of the total steady-state Ca^2+^ currents of hilMFBs and caMFBs. Note the smaller total current in hilMFBs as presented in independent measurements (see Fig. 3).

To ensure fast application and stable recordings, Conotoxin-GVIA or Agatoxin-IVA were applied locally via a pipette placed close to the recording site. Similar to earlier data (Pelkey et al., 2006; Li et al., 2007; Chamberland et al., 2017), the P/Q-type channels account for a greater proportion of the total calcium currents in MFBs than the N-type channels (Fig. 4*A–C*). Importantly, the proportion of both Conotoxin-GVIA-sensitive and Agatoxin-IVA-sensitive currents were similar in hilMFBs and caMFBs in separate experiments. These results suggest that the contribution of P/Q-type and N-type channels to the total Ca^2+^ currents are similar in hilMFBs and caMFBs. The similarity of the voltage-dependence of the total Ca^2+^ currents of hilMFBs and caMFBs (Fig. 4*D*) further supported the observed pharmacological results. Specifically, incremental voltage-steps revealed similar relative steady state current amplitudes, despite the different amount of the currents (half activation voltage, hilMFB: −15.58 ± 0.95 mV and caMFB: −16.22 ± 0.45 mV).

### Activity-dependent plasticity of AP shape and Ca^2+^ currents

**Figure 5.**
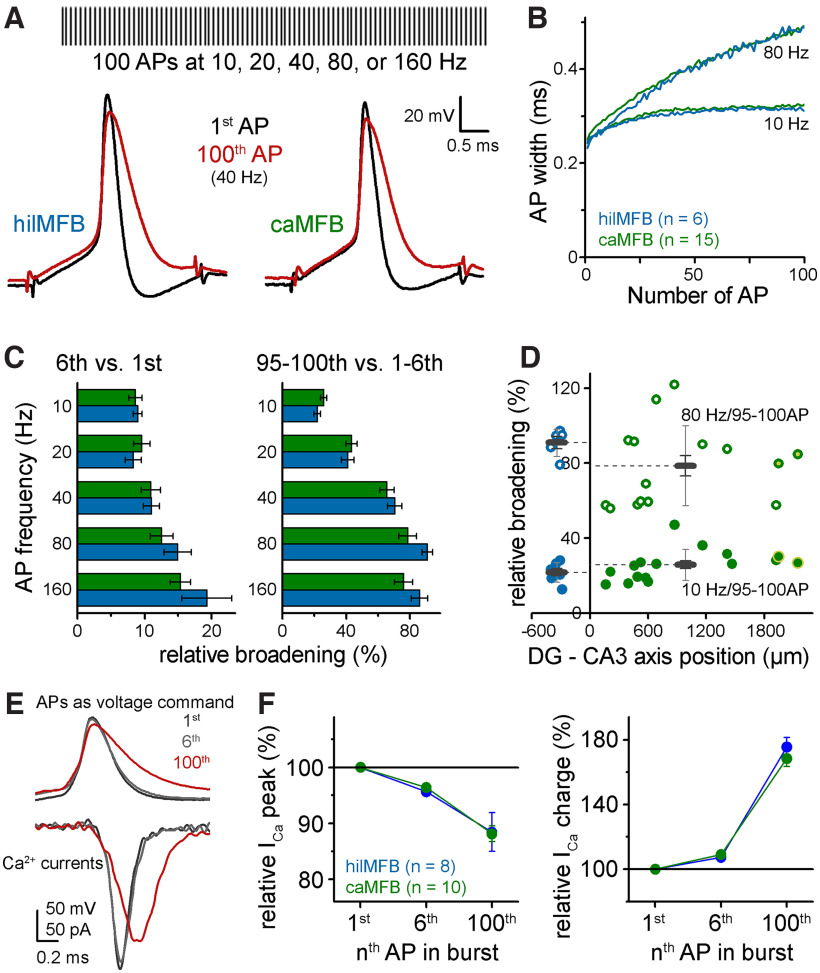
Similar AP broadening in hilMFBs and caMFBs in wide frequency ranges. ***A***, Schematic representation of AP trains, which were elicited with brief current pulses at 10, 20, 40, 80, and 160 Hz. Traces below show APs of a representative hilMFB and caMFB at the beginning and the end of 40-Hz trains. ***B***, Average half-widths during trains at two representative frequencies from all recorded caMFBs and hilMFBs. ***C***, Relative broadening of the width of 6th (left) and 95–100th (right) APs compared with the initial APs at various frequencies. ***D***, Dependence of the broadening on the relative position of the recorded MFBs. Only the 10- and 80-Hz data are shown where the width of the 95–100th APs were compared with 1–6th APs. ***E***, APs applied as voltage waveforms in voltage clamp recordings to measure activity-dependent changes of the elicited Ca^2+^ currents. ***F***, Peak and charge of Ca^2+^ currents evoked by voltage waveforms of the 6th and 100th APs of the 40-Hz train relative to those evoked by the first APs.

Action potentials in MFBs show activity-dependent plasticity that dynamically changes the synaptic effect of individual action potentials (Geiger and Jonas, 2000). Specifically, the width of APs widens during high frequency trains. This widening is thought to contribute to the characteristic facilitation of synaptic release from MFBs, since wider APs elicit larger Ca^2+^ influx. Therefore, next we compared the plasticity of APs in hilMFBs and caMFBs during train activation. Likewise in previous studies (Geiger and Jonas, 2000), we stimulated whole bouton recorded MFBs with 100 APs at 10, 20, 40, 80, and 160 Hz and quantified AP widths during these trains (Fig. 5*A*,*B*). These frequencies occur in GCs during short physiological bursts (Buzsáki et al., 1983; Jung and McNaughton, 1993; Diamantaki et al., 2016; GoodSmith et al., 2017; Senzai and Buzsáki, 2017) and have a remarkable effect on MF synapses (Neubrandt et al., 2018). We compared broadening at the beginning of these trains (1st vs 6th APs), which refers to changes during physiological bursts, and during longer trains (1–6th vs 95–100th APs), which were used previously to study AP broadening in MFs and other axon types as they nearly maximize this plasticity phenomenon (Geiger and Jonas, 2000). Consistently with previous studies, MFBs showed substantial AP broadening even during short bursts, and the broadening was dependent on both the frequency and the duration of activity (Fig. 5*B*,*C*). However, no considerable difference was found between this form of AP plasticity in hilMFBs and caMFBs. Furthermore, AP dynamics showed no correlation to the location of the MFB along the DG-CA3 axis (Fig. 5*D*). Thus, we concluded that similar to basic properties of AP-I_Ca_ coupling, the plasticity of APs is similar in hilMFBs and caMFBs.

Finally, we tested how Ca^2+^ influx follows activity-dependent AP shape changes in the two populations of MFBs, which can mediate differential AP-release coupling during bursts (Geiger and Jonas, 2000; Bischofberger et al., 2002; Drakew et al., 2017). For this aim we measured isolated calcium currents in voltage clamp recordings and employed naturally propagating AP waveforms as voltage commands. Specifically, we used the first, sixth and hundredth APs from a 40-Hz train, which show progressive broadening and a slight decrease in the amplitude (Fig. 5*E*). Similar to previous reports (Geiger and Jonas, 2000; Bischofberger et al., 2002), broader MFB APs evoked Ca^2+^ currents with smaller peak amplitude. Furthermore, as expected from the smaller driving force change from the slower repolarization, Ca^2+^ currents peaked later during broader APs. Thus, because of the slower kinetics, the overall charge was larger, which may contribute to the synaptic facilitation (Geiger and Jonas, 2000). Evidently, similarly to other stages of AP-I_Ca_ coupling we found no differences between the AP-shape dependent Ca^2+^ current dynamics of hilMFBs and caMFBs (Fig. 5*F*).

Thus, these results suggest that, in addition to the basal properties of AP-I_Ca_ coupling in hilMFBs and caMFBs, they are subject of the same activity dependent plasticity mechanisms that result in similar changes in presynaptic Ca^2+^ signals during physiological activity patterns.

## Discussion

Here, we showed that the key steps of AP-I_Ca_ coupling are indistinguishable in the two populations of large MFBs that target PCs and MCs. This observation suggests that the different cell type identities of CA3PCs and MCs are not supported by a different presynaptic module of their main excitatory input. To elaborate, first we showed that the properties of local APs are similar in hilMFBs and caMFBs. Thus, both synapse types perceive the same digital activity of the same presynaptic source. Interestingly, the AP-Ca coupling is indistinguishable despite the small difference in the average sizes of the hilMFBs and caMFBs (Claiborne et al., 1986; for further discussion, see below). Not only the two region-defined MFB populations were similar, but we did not observe distance dependent differences in the AP parameters of caMFBs along the proximo-distal axis either. This suggests that the potential functional differences of CA3PCs along the CA3 axis (Lee et al., 2020) is not because of a gradient in an AP-I_Ca_ coupling function. Next, we demonstrated that the kinetics and voltage dependence of the Ca^2+^ currents in hilMFBs and caMFBs were indistinguishable. It is worth noting that the amplitude of Ca^2+^ currents were larger in caMFBs compared with hilMFBs. However, all large MFBs incorporate a high number of release sites with compartmentalized and relatively independent Ca^2+^ signaling (Chamberland et al., 2018); therefore, if we consider this shared anatomic feature simultaneously with the size differences between caMFBs and hilMFBs, then we can conclude that the relative density of the current is of greater importance for the AP-I_Ca_ coupling. Indeed, the bouton-size normalized Ca^2+^ amplitude, or current density, was not significantly different between hilMFBs and caMFBs, and did not show any gradient along the CA3 axis. Using two independent datasets, which employed either conventional square steps or native AP waveforms as voltage commands, similar Ca^2+^ current densities in hilMFBs and caMFBs was seen. Furthermore, Ca^2+^ currents were similar not only at macroscopic levels, but also at molecular level, as they were mediated by a similar set of P/Q-type and N-type channels both in hilMFBs and caMFBs. Our results confirm previous findings of the dual contribution of these channel types in caMFBs, which further suggest that similar effectors are available for presynaptic modulatory pathways (Pelkey et al., 2008; Brunner et al., 2013). Finally, we revealed that similar plasticity mechanisms modulate APs in hilMFBs and caMFBs during repetitive GC activity. Specifically, APs of hilMFBs showed similar frequency-dependent broadening as described in caMFBs during sustained firing between 10 and 160 Hz (Geiger and Jonas, 2000). Considering the similar contribution of N-channel and P/Q-channel types to the total Ca^2+^ current evoked by square pulses and AP-waveforms, it was not surprising that the dynamically changing AP waveforms elicit similar Ca^2+^ influx. Thus, the plasticity of AP-I_Ca_ coupling does not result in distinct signals for CA3PCs and MCs during repetitive GC activities.

Obtaining good quality current clamp data from axon terminals is difficult not only because of their small size, but also the potential interference of the instrumental capacitance with the small structure (Olah et al., 2021). Therefore, it is important to emphasize that the sizes of hilMFBs and caMFBs are in an overlapping range where this potential interference would be similar and therefore the potential effect of it is minimal. Furthermore, the independent voltage clamp measurements also revealed size-independent current properties.

The major conclusion of this work is that CA3PCs and MCs share their main presynaptic module. Thus, they read the same DGGC activity identically. This modular establishment can be related to the recent functional divergence of these two cell types (Blackstad and Kjaerheim, 1961), and it certainly has an economical benefit for the circuit. On the other hand, the similarity of AP-I_Ca_ coupling is not simply because of the shared presynaptic source, because MF synapses are known to show large diversity. The same axon form morphologically distinct boutons (Amaral, 1978; Acsády et al., 1998) onto pyramidal cells and GABAergic cells, which elicit diverse synaptic responses in terms of release properties (Toth et al., 2000; Szabadics and Soltesz, 2009; Torborg et al., 2010). Importantly, the shared components in distinct cell types should be also considered when specific interventions are designed to tackle mis-functions of distinct cell types. For example, reinstating the normal contribution of MC functions in epilepsy treatments should preferentially avoid targets that involve the AP-I_Ca_ coupling of their main excitatory drive from MFs, because such intervention will also impact the essential memory functions of the CA3 circuit.

These results imply that the division line of MCs and CA3PCs is thin. But other mechanisms account for their different functions, which are independent from their input from DGGCs. Only CA3PCs receive direct input onto their distal dendritic tuft from the entorhinal cortices in a well-organized layer of the stratum lacunosum-moleculare and local inhibitory inputs also differs (Larimer and Strowbridge, 2008; Sun et al., 2017b). Both CA3PCs and MCs form recurrent innervation to their own groups of cell types (Miles and Wong, 1986; Amaral et al., 1990; Wittner et al., 2007; Wittner and Miles, 2007; Larimer and Strowbridge, 2008; Sun et al., 2017b); however, the abundance and functions of these connections were not compared. There are also key differences in the output of MCs and CA3PCs. The main target zone of MCs is the proximal dendritic region of DG granule cells, and a single MC usually innervates a very large portion of both the ipsilateral and the contralateral hemispheres. Whereas, CA3PCs form an extensive recurrent network which is considered as a substrate of memory storage and recall. In addition to the similarity of their main excitatory inputs from DGGCs, several other properties are shared by MCs and CA3PCs at the level of their morphology, excitability and molecular content (Buckmaster et al., 1993; Cembrowski et al., 2016). Our results argue that relatively few differences destine MCs and CA3PCs into distinct cell types, while they use identical molecular modules for shared functions, which requires only fine-tuning for the specific functions of the cell type. Our findings reveal that the AP-I_Ca_ coupling of their main synaptic drive from DG GCs also belongs to the shared neuronal instruments of CA3PCs and MCs.

In addition to the main general conclusion of these results, several points can be made. First, the AP-I_Ca_ coupling at MFs does not show soma-synapse distance-dependent functions; either between the hilus and CA3 regions, and within the CA3 region. Thus, the previously observed gradient in the strength of MF-evoked responses along the CA3-CA2 axis (Sun et al., 2017a) should be mediated by the synaptic machinery downstream of the Ca^2+^ influx, which may also contribute to the differential involvement of proximal and distal CA3 regions to pattern separation and pattern completion (Lee et al., 2020). However, in case of the CA2 pyramidal cells, the smaller size of the boutons (Kohara et al., 2014), and the consequent lower number of release sites, may also contribute to the weak MF evoked responses. Second, our results also suggest that MFBs that contact pyramidal cells in the CA2 region use the same AP-I_Ca_ coupling mechanisms as CA3PCs and MCs. Interestingly both MFBs onto CA2PCs and MCs are smaller than those contacting CA3PCs (Claiborne et al., 1986; Kohara et al., 2014), which was also reflected in our membrane capacitance and input resistance measurements. On the other hand, the membrane time constant, AP parameters and dynamics were similar in MFBs within the CA3 and CA2 regions. Although, passive parameters can be explained by the different size of the hilMFBs and caMFBs, we cannot exclude the possibility that differential Kv7 functions also contribute to their resting states. However, the similar resting membrane potential and AP threshold values suggest that Kv7 function is rather similar in the two regions, which implies that these channels have similar contribution to shaping presynaptic Ca^2+^ influx (Martinello et al., 2019). Nevertheless, activity-dependent changes in focal cholinergic tone may contribute to the functional diversity of the excitability of MF terminals (Martinello et al., 2015). Third, an additional consequence of the smaller size of hilMFBs was the smaller total Ca^2+^ current compared with caMFBs. However, the densities of Ca^2+^ currents, which normalize the current amplitude to the membrane capacitance of individual boutons, were again very similar in hilMFBs and caMFBs. Considering the smaller number of release sites in the smaller hilMFBs, the above results suggest that the number of Ca^2+^ channels and Ca^2+^ influx are also similar at the level of individual release sites.

Although it is likely from their indistinguishable properties and shared presynaptic source, it remains an open question whether MCs and CA3PCs employs the same set of trans-synaptic adhesion molecules for the establishment of MFBs and thorny excrescences (de Wit and Ghosh, 2016). Finally, it should be emphasized that despite the shared cellular devices, such as the shared AP-I_Ca_ coupling mechanisms described here and morphologic features, the distinct functionality of MCs and CA3PCs are supported by a number of fundamental features unique to each, including synaptic projections to different targets and distinct input sources. Future studies should reveal the specific contribution of these functionally defying mechanisms, especially for therapeutic interventions when MCs or CA3PCs are needed to be targeted selectively.

## Synthesis

Reviewing Editor: Viji Santhakumar, University of California Riverside Decisions are customarily a result of the Reviewing Editor and the peer reviewers coming together and discussing their recommendations until a consensus is reached. When revisions are invited, a fact-based synthesis statement explaining their decision and outlining what is needed to prepare a revision will be listed below. The following reviewer(s) agreed to reveal their identity: NONE. Note: If this manuscript was transferred from JNeurosci and a decision was made to accept the manuscript without peer review, a brief statement to this effect will instead be what is listed below.

This is a transfer from J.Neurosci with excellent reviews. The original submission was recommended for rejection based only on the negative results. No scientific or methodological concerns were noted and reviews were uniformly laudatory.
